# Study of Acid Whey Fouling after Protein Isolation Using Nanofiltration

**DOI:** 10.3390/membranes11070492

**Published:** 2021-06-30

**Authors:** Marjana Simonič, Zorka Novak Pintarič

**Affiliations:** Faculty of Chemistry and Chemical Engineering, University of Maribor, Smetanova 17, SI-2000 Maribor, Slovenia; zorka.novak@um.si

**Keywords:** acid whey, nanofiltration, fouling, Tansel, Hermia

## Abstract

In this paper, nanofiltration (NF) of acid whey after isolation of proteins was studied. Two membranes were tested: NF-99 (Alfa Laval) and DL (Osmonic Desal). Based on previous measurements that determined the highest efficiency in separating lactic acid and lactose whey, the pH was adjusted to 3. First, the most appropriate transmembrane pressure (TMP) was determined based on the highest flux measured. The TMP range was 5–25 bar for the DL membrane and 10–30 bar for the NF-99 membrane. The temperature was kept at 4 °C using a thermostat. The mechanisms of membrane fouling were investigated. The Hermia models and the modified Tansel model were applied to study the fouling mechanism and to determine which membrane would foul earlier and more severely, respectively. The most suitable TMP was determined at 20 bar. Despite the 1.4 times higher flux of the sample at DL, the fouling rate was higher when NF-99 was used. The results showed that the Tansel model is suitable for predicting the fouling time of protein-isolated whey by nanofiltration.

## 1. Introduction

Whey is divided into sweet whey, which is a by-product of cheese production, and acid whey, from the production of fresh and cream cheese, Greek Yogurt, and caseinates [1]. There is an interest in new applications of whey and its derivatives [2]. Lactose constitutes the major 5–6% of whey and can be used to produce glucose and galactose by hydrolysis.

The disposal of acid whey is complicated due to its high biological oxygen demand and high organic matter content (COD), resulting in the need for costly water treatment before discharge into the environment [1]. Bioeconomy is very important in terms of waste management [3]. It has been shown that waste is a valuable resource, and food waste requires sustainable management to reduce its hazardous impact on the environment and add value for a better economy [4]. Upflow Anaerobic Sludge Bed (UASB) treatment of food waste leachate achieved a 36% improvement in effluent COD. Several methods are available for the concentration of whey, such as membrane technologies [2]. Most researchers have focused on protein fractionation. Ultrafiltration has been used to fractionate proteins from whey [5]. The permeate was concentrated with NF, and the lactose content was increased by NF application with increasing operating temperature. Protein lactoferrin was isolated from acid whey obtained from the production of fresh curd cheese [6]. The results showed the high potential of monolithic ion-exchange chromatography for industrial processing of acid whey as a source of lactoferrin. The protein products have high added value.

Nanofiltration (NF) is considered to have great potential for food processing applications, such as dairy processing [7]. Nanofiltration membranes could be used, for example, to separate lactose from whey [8]. The best results have been obtained at a transmembrane pressure (TMP) of 20 bar. Fouling problems were not found in the laboratory NF tests performed, but the economic feasibility of such a nanofiltration process was not evaluated. Recently, the combination of NF and electrodialysis was shown to provide better quality of the final powder product when using spray drying [9]. Up to 88% of the lactic acid was removed and the product contained less water after all applied methods. A higher content of water, minerals, and organic acids leads to particle aggregation.

In general, membrane fouling can be a significant problem in the use of nanofiltration for whey treatment. The main contributors to membrane fouling are calcium phosphate salts that interact with whey proteins [10]. At higher temperatures, proteins naturally tend to precipitate (e.g., 50 °C), and such calcium-based protein interactions can enhance membrane fouling. Aggregates with a diameter smaller than that of the pores could become bound to the inner membrane pore side and block membrane flux [11]. Protein cross-linking due to the formation of calcium bridges and Ca_3_(PO_4_)_2_ precipitation are involved in membrane fouling. At higher temperatures, calcium phosphate becomes increasingly insoluble [12]. Since less than 20% of all proteins in whey remain in the flow-through, the expected fouling rate is much lower [6]. Therefore, nanofiltration could be used to treat whey after fractionation of proteins.

It has been found that the operating parameters affecting membrane performance are membrane chemistry, transmembrane pressure (TMP), pH, and solute concentration of the feed, e.g., whey [13]. The best results for oligosaccharide isolation using NF were obtained at 20 bar. NF has been used for the treatment of acidic mine drainage [14]. The results showed that the DL membrane is preferable for a high concentration of acidic main drainage; on the other hand, NF99 is used when high permeate flux is required. Experiments were carried out at TMP between 20 and 30 bar. In another study, the best operating conditions were also determined at TMP of 20 bar [15]. They studied the organic content expressed as the chemical oxygen demand (COD). The COD was measured below 5 g/L after NF application.

The main objective of the present research was to study the fouling properties of selected nanofiltration membranes DL and NF-99 in the treatment of acid whey, after fractionation of proteins. The aim of the research was to determine optimal conditions for the nanofiltration of lactic acid whey using both membranes. To the best of our knowledge there is a lack of modeling regarding NF of acid whey. The modified Tansel model was used to predict membrane fouling and verified with experimental results obtained using a laboratory NF system. Nonlinear fitting was performed using the modeling system and optimization solvers in the software GAMS (www.gams.com accessed on 29 June 2021).

## 2. Materials and Methods

**Figure 1:**
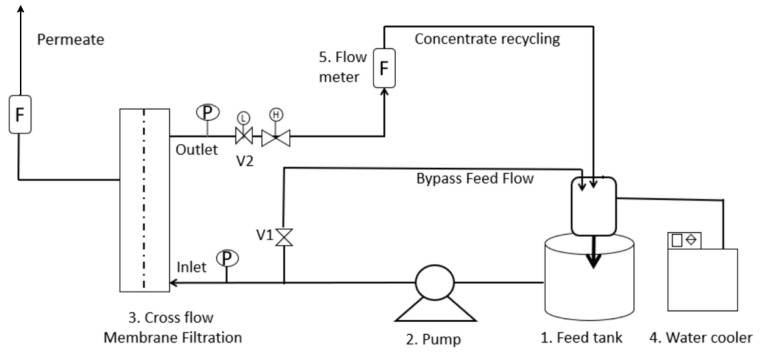
A schematic of the NF process.

Acid whey (Dairy Celeia, Petrovče, Slovenia) was filtered using ceramic asymmetric multichannel alumina/zirconia membranes with a pore diameter of <0.8 μm in a tangential filtration system (JIUWU HI-TECH, Pukou, Nanjing, Jiangsu, China). Proteins were isolated by monolithic ion-exchange chromatography. The remaining flow-through fraction (FT) was further treated in the MemCell, Osmo membrane system (Korntal-Munchingen, Germany) shown in Figure 1.

The volume of the feed tank was 2 L. Feed solution (FT) flowed from the feed tank through the valve and pump to the membrane cell. The system was equipped with flow meters (F) and pressure meters (P). The permeate was continuously withdrawn from the system, and the concentrate was returned to the feed tank. The transmembrane pressure (TMP) ranged from 10 to 30 bar for the NF-99 and from 5 to 25 bar for the DL membrane. The water permeate flux was measured for each 5 bar increase in transmembrane pressure for approximately 30 min. In the next step, FT was filtered at the TMP where the water flux was the highest. Each trial of FT treatment was performed at a constant temperature of 4 °C using a thermostat and repeated three times. The permeate and feed samples were taken at the end of the trial and subsequently analyzed. After every repeated trial with FT nanofiltration, the water flux was measured for fouling calculations.

NF-99 and DL (Alfa Laval) membranes with the properties listed in Table 1 were used. The effective membrane area was 0.08 m^2^.

Analyses of FT samples and permeate were performed according to ISO standards in three replicates. The standard methods are summarized in Table 2. Absorbance at 436 nm was measured as an indication of inorganic contamination. The sum of all organic compounds in the flow-through fraction was determined as the chemical oxygen demand (COD).

### 2.1. Zeta Potential Measurements

The zeta potential was measured using a cylindrical cell within an electrokinetic analyzer (SurPASS, Anton Paar GmbH, Graz, Austria). Membranes were wetted with 0.001 M KCl solution, which was also used as the background electrolyte. The pH dependence of the zeta potential in the range pH 3–9 was determined using 0.1 M NaOH as the titration liquid. The zeta potential was calculated from the measured streaming flow using the Helmholtz–Smoluchowski equation [16].

The zeta potential of FT was determined by the dynamic light scattering technique using the Malvern Zetasizer instrument (Malvern Instruments, Malvern, UK).

Reversible fouling F_r_ (-) was determined according to Equation (1):

F_r_ = (J_w_ − J_s_)/J_w_ (1)

Irreversible fouling F_ir_ (-) was determined according to Equation (2):

F_ir_ = (J_w_ − J_wf_)/J_w_ (2)

where

J_w_ = water flux through virgin membrane (L/(m^2^h));

J_wf_ = water flux through membrane after sample filtration (L/(m^2^h));

J_s_ = sample flux (L/(m^2^h)).

The decrease in permeate flux during the NF process is a confirmation of the fouling phenomenon, which can be caused by the formation of a cake layer, and various types of plugging of membrane pores according to Hermia models [17,18]. The decrease in permeate flux during the NF process is a sum of contributions originating from the mechanisms defined by Equations (3)–(6), summarized in Table 3.

J and J_o_ are the final and initial flux (L/(m^2^h)), *K* represents a constant for each model, and t is the filtration time (min). From linear plots, the constant *K* was determined as the slope of the line. In addition, regression coefficients (R^2^) were determined from the above models.

The modified Tansel’s first-order kinetic model (Equation (7)) has previously been used to predict critical fouling in dead-end filtration and cross-flow filtrations [19]. In this model, the total resistance is considered as a combination of time-independent resistance and time-dependent resistance. At the conversion time point, the resistance remains constant.

(7) $$\frac{1\;}{J}=a-{\mathrm{be}}^{-t/\tau }$$

Here, a represents the time-independent resistance (m^2^h/L), the coefficient b (m^2^h/L) stands for the time-dependent resistance, and the coefficient τ is the fouling time constant (min).

### 2.2. Nonlinear Regression

A regression model to fit the experimental data to a nonlinear equation was programmed in the General Algebraic Modeling System (GAMS). The model is based on minimizing the squares of the differences between the measured and calculated values. A CONOPT solver was used to solve the model.

## 3. Results and Discussion

### 3.1. Physico-Chemical Analyses

After all proteins were isolated from the original whey sample, some general parameters were measured in FT and NF permeates, as shown in Table 4. The COD of FT exceeded 60 g/L O_2_. The pH was adjusted to 3.3 with 0.1 M HCl, conductivity (before adjustment) was 7.35 mS/cm, and a turbidity of 1280 NTU was determined in FT. Analyses after NF-99 filtration showed that the turbidity decreased to 0.9 NTU, and we assume that most of the fats were removed. The pH value remained almost unchanged. The absorbance at 436 nm decreased by 98%.

Although the COD removal efficiency was around 98%, the values in the permeate streams were still relatively high. According to the Slovenian regulation [20], the limit COD value for discharge into the sewerage system is 0.125 g/L O_2_. It can be seen from Table 4 that the COD values of the permeates exceeded the permissible value by more than 10 times. Thus, they cannot be discharged to the environment without additional treatment. The measured values were comparable to those in a study where the concentrations of treated whey after NF decreased below 5 g/L and 1 g/L O_2_ at TMP of 20 bar and 10 bar, respectively [15].

**Figure 2:**
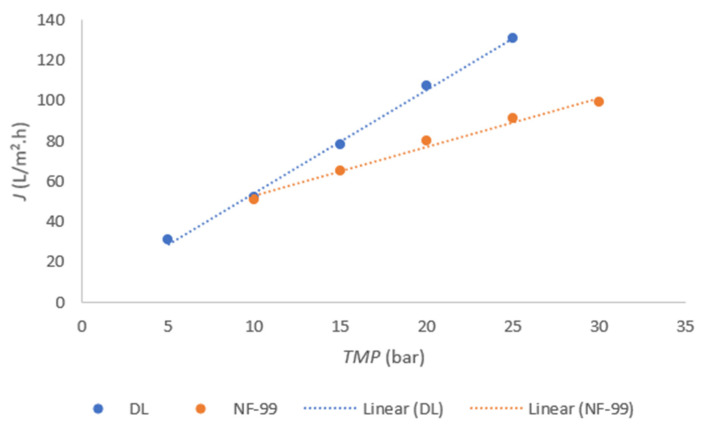
Flux depending on the TMP for the DL and NF-99 membranes.

First, the most favorable TMP was determined based on the flux measurement. The results of flux dependence on time are shown in Figure 2 for the DL membrane and NF-99 membrane.

The linear plot for permeability was determined using DL, as shown in Figure 2. Similar values were also obtained in another study [16]. The permeability of NF-99 is shown in the range from 10 bar to 30 bar, since the flux was very low at TMP of 5 bar. A small deviation was observed at 20 bar with the NF-99 membrane, and a similar deviation, but slightly smaller, was also observed with DL, indicating that the highest flux was reached at 20 bar TMP. At higher TMP, the flux tends to decrease. Therefore, 20 bar TMP was chosen for further trials with both membranes. The same TMP was previously reported to be optimal for the nanofiltration of acid whey [1].

### 3.2. Membrane Fouling Study

#### 3.2.1. Zeta Potential

**Figure 3:**
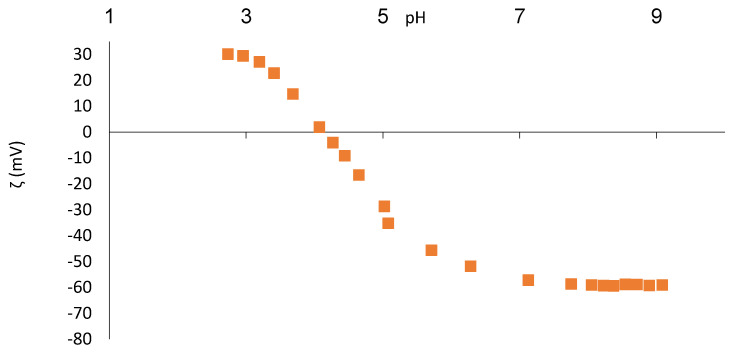
Zeta potential of the clean DL membrane as function of pH.

**Figure 4:**
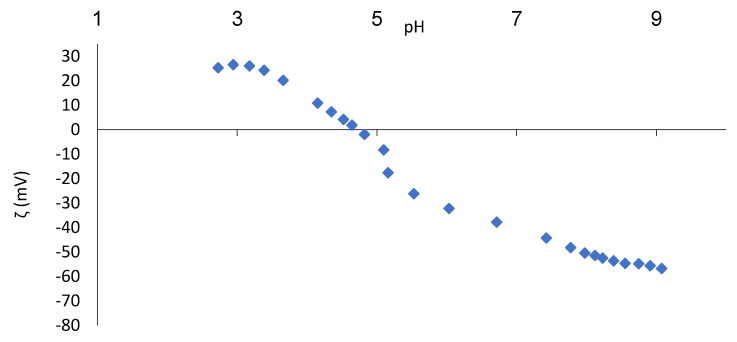
Zeta potential of the clean NF-99 membrane as function of pH.

Zeta potential data as a function of pH for the experiments performed are shown in Figure 3 and Figure 4 for both membranes. The DL membrane has an iso-electrical point (IEP) at pH = 4.1, and NF-99 has the same at pH = 4.7. For both membranes, the surface charge is positive in the lower pH range, passes through the IEP, and then becomes negative in the upper pH range.

The IEP for nanofiltration membranes is mostly found in the pH range of 3 to 6 [16]. For the Filmtec NF245 (Dow Chemicals, Midland, MI, USA) membrane, an IEP close to 4 has been reported [21]. In other studies, the IEP for the NF-99 membrane was measured at pH = 4.1–4.4 [22] and that for DL was measured at pH = 2.7 [16] and pH = 4.0 [23]. However, it is very important that we know the electrolyte used, as the IEP can vary depending on the present ionic strength [21]. Figure 3 shows that a constant value of zeta potential is already reached at pH = 8, while the zeta potential of the NF-99 membrane still slightly decreases above pH = 8. A comparison of the zeta potentials of both membranes shows a similar maximum zeta potential value at about −60 mV in both cases. However, the charge at acidic pH is more important for nanofiltration of FT. Both membranes have a positive zeta potential during nanofiltration of whey at pH = 3. After nanofiltration of FT, the zeta potential curve remains very similar to that of the unfouled membrane and practically overlaps with the zeta potential curve of the clean membranes in both cases.

The zeta potential of FT was determined between −2 and −3 mV, matching the imperceptible changes in the IEP of the two fouled membranes. The unchanged IEP after nanofiltration indicates that the fouling material is not charged or is only very slightly charged [24], which was the case in the present FT study.

#### 3.2.2. Membrane Flux Measurements

**Figure 5:**
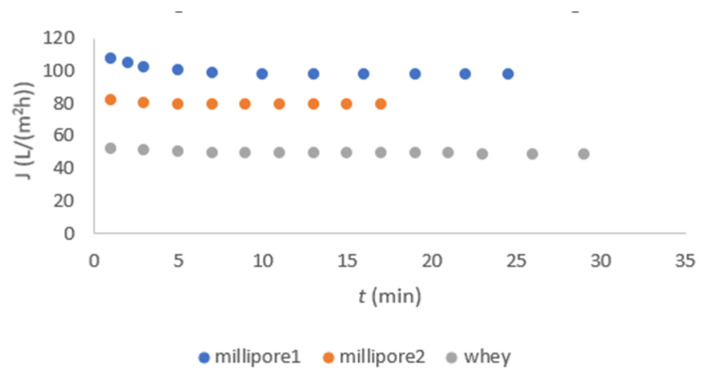
Flux dependent on time for the DL membrane.

**Figure 6:**
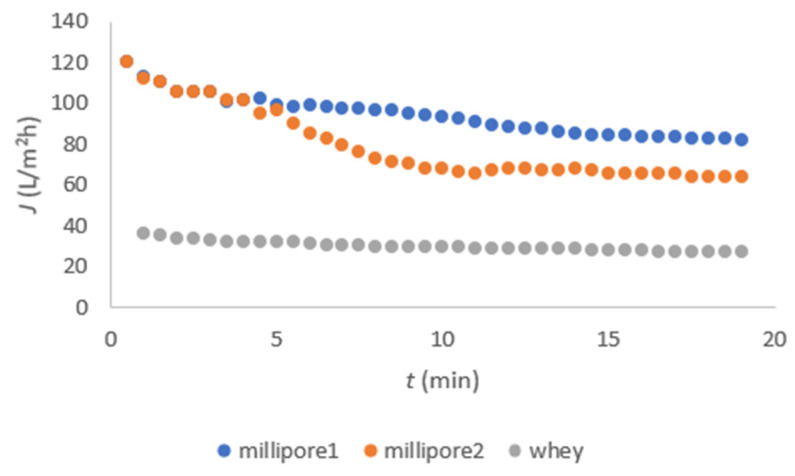
Flux dependent on time for the NF-99 membrane.

The fluxes of millipore water before and after FT nanofiltration, as well as that of FT nanofiltration itself, are shown in Figure 5 for DL and in Figure 6 for NF-99. These data are important for calculating the reversible and irreversible fouling of the membrane.

In Figure 5, a relatively uniform flux was observed due to the removal of fats, proteins, and solids from the acid whey by microfiltration pretreatment. The same observation was reported by Chandrapala et al. [1]. The flux of FT was significantly higher in DL nanofiltration as compared to NF-99. The millipore water flux was determined at TMP of 20 bar using both membranes. The flux was higher when using the DL membrane. The experiments were stopped after 20 min as the temperature started to rise above 280 K. It was assumed that the activity of lactic acid bacteria increases, and they start to produce lactic acid from lactose, which was contrary to the aim of our study to separate lactose from lactic acid [6].

#### 3.2.3. Membrane Fouling Studies and Modeling

The reversible and irreversible fouling of the two NF membranes was determined at 20 bar TMP using Equations (1) and (2). The results are presented in Table 5.

It was found that irreversible and reversible fouling was lower when tested with DL. Thus, DL is superior to NF-99 due to its lower fouling rate; consequently, chemicals are saved and the environment is protected due to less pollution with less chemicals used for cleaning. In addition, the flux of FT is almost 2 times higher when a DL membrane is used.

**Figure 7:**
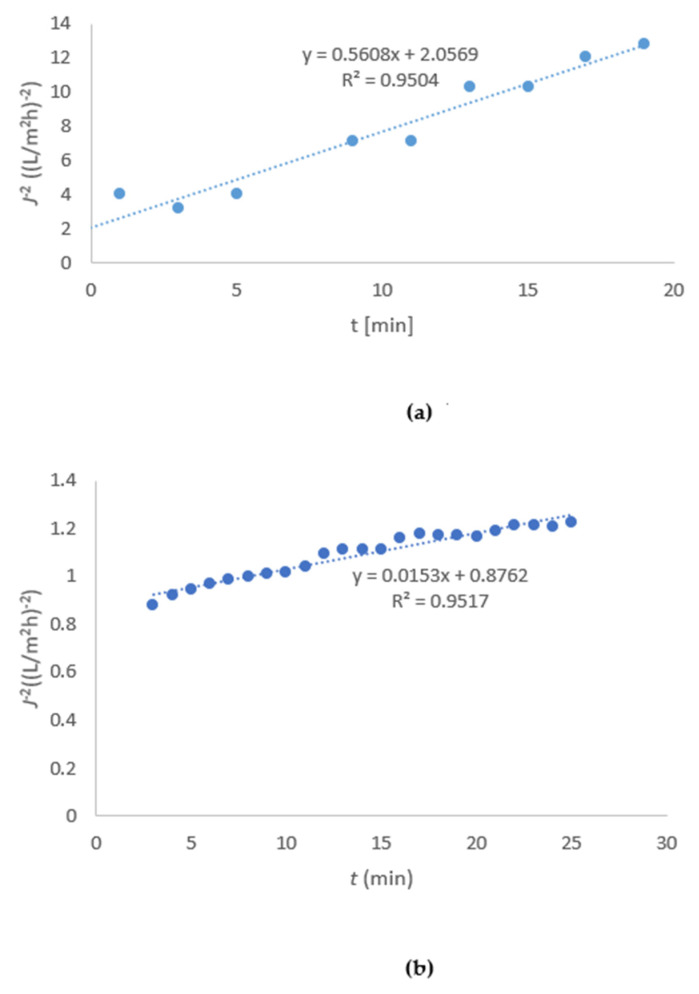
Flux dependent on time representing the cake layer formation model for the DL (**a**) and NF-99 (**b**) membranes.

Experiments were continued at 20 bar to determine the model of clogging. The curve *J*^−2^ as a function of time was determined according to the result of Equations (3)–(6). R^2^ was highest when the cake layer formation model was used (Equation (6)), so only this type of curve is shown (others not shown). In Figure 7a, it can be seen that the slope *K* was determined to be 0.56 and *R*^2^ was 0.95 in the case of the DL membrane. In Figure 7b, it can be seen that the slope *K* for the NF-99 membrane was determined to be 0.015 and *R*^2^ was 0.95. The fit to a linear plot was best when using Equation (6) for both membranes, indicating the formation of a cake layer.

The cake layer forms via the deposition of material on the membrane surface, rather than by penetration into the membrane pores [17]. On the other hand, the same authors reported that pore blocking may form at the pore entrance, or the pore may be completely blocked. Since the *R*^2^ values of the different types of pore blocking determined by Equations (3)–(5) in Table 3 were lower, it can be concluded that these fouling mechanisms affect the membrane to a lesser extent. The results are consistent with the calculations of F_ir_, which was much lower compared to reversible fouling; the latter is more easily removed from the membrane by back flushing.

In the next step, we fitted the experimental data to Equation (7) to investigate the relationship between time-dependent and time-independent resistance in membrane fouling. All three coefficients in Equation (7) were obtained by nonlinear fitting with experimental plots using GAMS. The values of *a*, *b*, and τ for both membranes are listed in Table 6.

An analysis of the coefficient values showed that the membrane characteristics mainly influence the values of the model parameters. Despite the higher flux with the DL membrane, the fouling rate was slower and lower compared to that for NF-99. In the case of NF-99, the flux of FT was lower, and the time-dependent resistance b increased, leading to an earlier reaching of the critical point. When the coefficient a is much larger than b, it indicates membrane resistance [25]. In our experiments with both membranes, the coefficient a was higher than b, but of the same order of magnitude, implying that fouling resistance is composed of membrane resistance and cake layer resistance. After a very short initial time, less than 1 min, a fouling layer formed. However, the fouling layer was loose and non-adhesive, as also reported in another study [25]. The initial phase, when only membrane fouling accounts for a major part of the fouling resistance, is very short. Cake layer fouling accounts for a large fraction of resistance in both membranes from almost the beginning of nanofiltration.

In FT, large concentrations of Ca, K, and Fe ions are still present [6], which contribute to inorganic fouling on the membrane surface. Fouling was alleviated more by the use of the DL membrane due to the intensification of inorganic fouling [19]. The results in Figure 5 and Figure 6 show that the FT flux was lower for the NF-99 membrane, which is probably due to stronger interactions of the inorganic compounds with the NF-99 membrane compared to DL.

**Figure 8:**
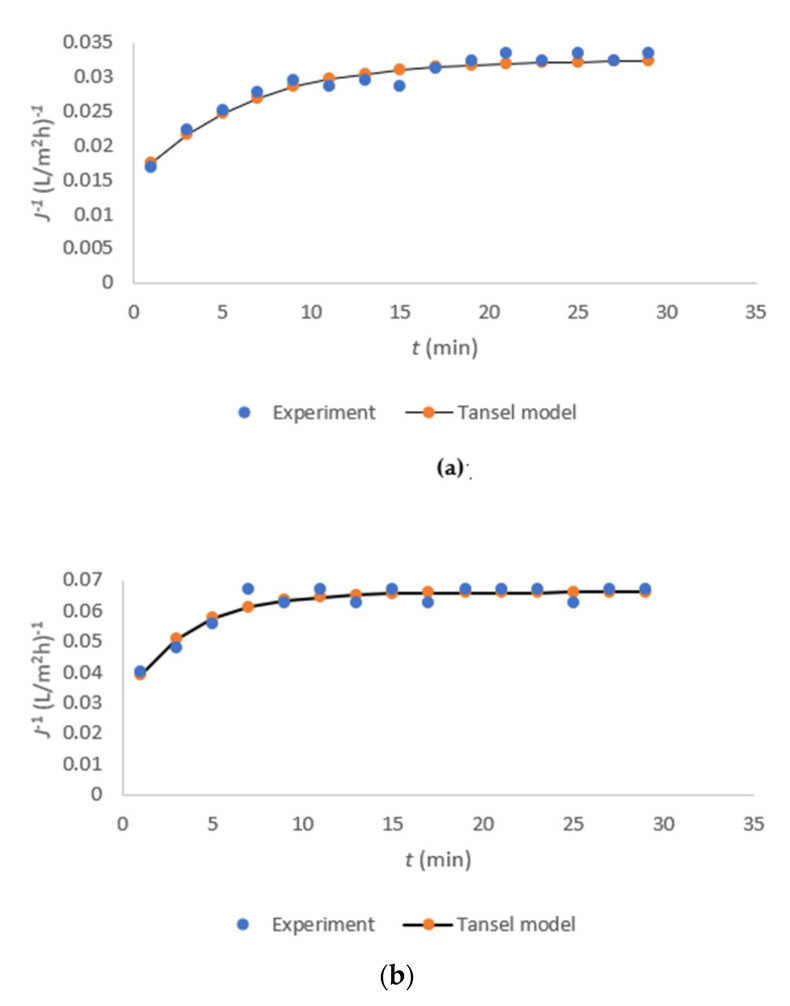
The results of nonlinear fitting with experimental plots for the DL (**a**) and NF-99 (**b**) membranes.

The results of nonlinear fitting with the experimental plots are shown in Figure 8a,b for the DL and NF-99 membranes, respectively. It is evident from Table 4 and Figure 8 that there is a very high matching with the modified Tansel model. Hence, it can be concluded that the modified Tansel model is suitable for describing the resistance during whey FT nanofiltration.

## 4. Conclusions

DL and NF-99 membranes were tested for predicting the fouling rate of whey pretreated with a ceramic membrane and monolith membrane for the fractionation of proteins. The optimum TMP was determined for both membranes at 20 bar. The experiments showed the superiority of the DL membrane for the treatment of pretreated whey after fractionation of proteins. The flux of the remaining flow-through fraction with the DL membrane was 60 L/(m^2^h), which is about 1.4 times higher than that when NF-99 was used. Despite the higher flux, the fouling rate was slower and lower compared to that for NF-99. These results could be explained using the modified Tansel and Hermia models in combination with the experimental data. The Hermia model showed that the predominant mechanism of fouling for both membranes was the formation of a cake layer, which was non-adhesive; furthermore, the fouling rate was slow. Using the Tansel model, the cake layer was found to form constantly, suggesting that time-dependent resistance was prevalent during filtration. The DL membrane could be used for a scaled-up process in a semi-industrial plant.

## Figures and Tables

**Table 1:** The NF membrane characteristics.

Parameter	NF-99	DL
Producer	Alfa Laval (Sweden)	Osmonic Desal (Germany)
P	1–55 bar	0.5–28 bar
T_max_	50 °C	50 °C
pH	2–10	2–11
MWCO	200 Da	340 Da
Morphology	Thin-film polyamide	Thin-film poly(piperazineamide)
Support	Polyester	Polyester

**Table 2:** The methods used for FT general chemical analyses.

Parameter	Standard Method	Apparatus
T (°C)	ISO 10523	Thermometer
pH	ISO 10523	pH meter, MA 5740
A (436 nm)	SIST EN ISO 7887	Spectrophotometer
Turbidity (NTU)	ISO 2027-1	Turbidity meter
COD (g/L O_2_)	ISO 6060	Digestion, titration

**Table 3:** The Hermia models [17,18].

	Equation		Plot
Intermediate pore blocking	J^−1^ = J_o_^−1^ – K⋅ t	(3)	t − J^−1^
Complete pore blocking	ln(J^−1^) = ln(J_o_^−1^) − K⋅ t	(4)	t − ln(J^−1^)
Standard pore blocking	J^−0.5^ = J_o_^−0.5^ − K⋅ t	(5)	t − J^−0.5^
Cake layer formation	J^−2^ = J_o_^−2^ − K⋅ t	(6)	t − J^−2^

**Table 4:** Measured chemical parameters.

Parameter	FT	NF-99 Permeate	DL Permeate
A(436 nm)	38.2	0.85	0.60
Turbidity (NTU)	1280	0.9	1.0
κ (mS/cm)	7.35	3.50	3.95
pH	3.3	3.2	3.2
COD (g/L O_2_)	60.3	1.5	1.9

**Table 5:** Reversible and irreversible fouling data.

Membrane	F_r_	F_ir_
DL	0.49	0.19
NF-99	0.67	0.21

**Table 6:** Coefficient values obtained by nonlinear fitting.

Coefficient	a (m^2^h/L)	b (m^2^h/L)	τ (min)	R^2^
Membrane DL	0.0324	0.01785	5.951	0.943
Membrane NF-99	0.0659	0.03628	3.466	0.908

## Data Availability

Not applicable.
